# Ecological Importance of Breeding Sites in Atlantic Forest Fragments: A Focus on Culicidae Diversity with Particular Attention to Vector Species

**DOI:** 10.1590/0037-8682-0108-2024

**Published:** 2024-12-16

**Authors:** Shayenne Olsson Freitas Silva, Cecilia Ferreira de Mello, Letícia de Mattos Pavão, Hélcio Reinaldo Gil-Santana, Jeronimo Alencar

**Affiliations:** 1 Instituto Oswaldo Cruz, Laboratório de Diptera, Rio de Janeiro, RJ, Brasil.; 2 Instituto Oswaldo Cruz, Programa de Pós-Graduação em Medicina Tropical, Rio de Janeiro, RJ, Brasil.

**Keywords:** Mosquitos, Traps, Breeding sites, Atlantic Forest remnant, Ecology

## Abstract

**Background:**

Vector distribution influences arbovirus persistence. This study examined the diversity of mosquito breeding sites in an Atlantic Forest fragment in Rio de Janeiro, Brazil.

**Methods:**

Mosquito specimens were collected at Fazenda dos Cordeiros, Silva Jardim, Brazil. Tire, plastic, bamboo, and sapucaia traps were evaluated for oviposition preferences using ecological indices.

**Results:**

Tire traps had the highest density. Bamboo traps showed the highest diversity. Plastic container was the most divergent site. The key recorded vector species included *Aedes albopictus* and *Haemagogus leucocelaenus*.

**Conclusions:**

Identifying the breeding sites that contribute the most to mosquito density is essential for optimizing control strategies.

Vector-borne diseases account for over 17% of all infectious diseases and result in more than 700,000 deaths annually. The etiological agents include parasites, bacteria, or viruses^1^. Mosquitoes have received considerable attention for their role in transmitting significant infectious pathogens, including arboviruses (e.g., Zika, dengue, chikungunya, and yellow fever) and parasites (e.g., malaria). These diseases pose a severe threat to global public health, particularly in tropical countries like Brazil^2^.

The persistence of arboviruses is primarily determined by the spatial and temporal distributions of their vectors, which are affected by several ecological factors^3^. Mosquito vectors thrive in a wide range of breeding habitats. Natural breeding sites include floods, floodplains, animal burrows, coconuts, shells, falling leaves, bamboo internodes, and bromeliads, which provide permanent or semi-permanent breeding grounds. Various physical, chemical, and biological factors can influence the selection, including light intensity or absence of light, color characteristics of the potential breeding site, temperature variations, salinity levels, the presence of plants or their products, microorganisms or their products, and substances associated with immature mosquito forms, among others^4^.

While many studies on mosquito breeding sites in the Atlantic Forest have focused on bromeliads^5^ or bamboos^6^, few have compared different types of breeding sites within the same area. Thus, this study aimed to compare the composition of mosquito species colonizing different breeding sites in a tropical forest remnant within the Atlantic Forest and to identify areas where species of public health concern can be found.

The permanent license for the collection, capture, and transport of zoological material was granted by the Chico Mendes Institute for Biodiversity Conservation (Chico Mendes de Conservação da Biodiversidade-ICMBio) and the Biodiversity Information Permission System (Sistema de Permissão de Informações sobre Biodiversidade-SISBIO) under N° 44333-1. All the members of the collection team were vaccinated against yellow fever. 

Collections were conducted on the border of Fazenda dos Cordeiros, located in the municipality of Silva Jardim, Rio de Janeiro state (22°36'32.5" S; 42°29'13.3" W) (Figure 1). This area maintains over 18.70% of the original coverage of the Atlantic Forest and consists mainly of a dense submontane ombrophilous forest^7^. 

**FIGURE 1:**
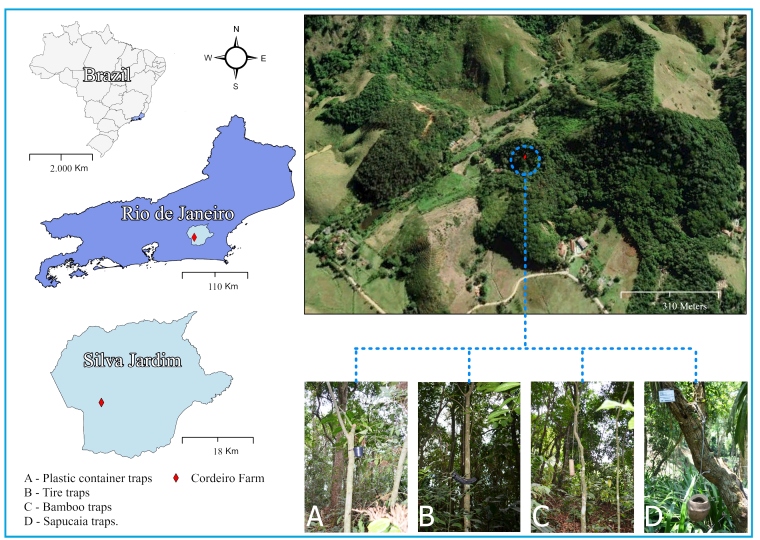
Map of the study site in the municipality of Silva Jardim, Rio de Janeiro State, Brazil. **Figure legends. A:** plastic container traps; **B:** tire traps; **C:** bamboo traps; **D:** sapucaia traps).

Monthly collections were performed from March 2021 to October 2023. Specimen collection consisted of setting up each trap vertically and fixing it to the trunks of four closely situated trees. The traps were positioned 2 m above the ground level. They were characterized as follows: a) Plastic trap: each trap consisted of a matte black pot with a 500 ml capacity, without a lid, to which 500 ml of natural water and leaf litter (the superficial layer of the forest formed by leaves, branches, and other organic materials) were added to reproduce an ecosystem similar to the natural one. b) Tire traps: This trap was made using 1/3 of a motorcycle tire with a 500 ml capacity. c) Bamboo trap: constructed from bamboo internodes separated to produce a container with a depth of approximately 30 cm, an opening approximately 25 cm in diameter, and a capacity of 500 ml. d) Sapucaia trap: This trap was made of woody fruit of the Brazil nut tree, with a diameter ranging from 12 to 20 cm, and a dehiscent lid or cap. For the experiment, a fruit capable of holding 500 ml of water was selected; the water in the trap occupied 2/3 of the fruit area.

Before installation in the field, traps were cleaned with a sponge and neutral soap, and tires were flamed to remove potential contaminants that could interfere with the efficacy of the trapping methods. The traps were replaced monthly. The collected specimens were individually removed, transferred to transparent bags, and transported to the laboratory. Subsequently, the traps were cleaned and prepared for field collection. 

The larvae and pupae captured in the traps were collected using a pipette and then transferred into 250 ml plastic bags (Whirl-Pak® bags, BioQuip®) for transportation to the laboratory. In the laboratory, the specimens were kept alive in pots containing water from the reservoir from which they were collected. Distilled water was added during evaporation. The immatures were reared until they reached the adult stage. During rearing, they were maintained under controlled thermoperiod and photoperiod with a temperature of 28 ± 1 °C, relative humidity of 75% to 90%, and a photoperiod of 12h. The larvae were fed with TetraMin® fish food, which was crushed and diluted in water.

All traps (tire, bamboo, plastic, and sapucaia) were immersed in plastic basins filled with distilled water to hatch eggs that could have been deposited on the walls of the traps. Immatures that reached the adulthood stage were killed by intoxication with a solution of ether or chloroform.

Adult mosquitoes were identified through direct observation of their morphological characteristics under a stereomicroscope (Zeiss New York, NY, USA) using the dichotomous keys described by Forattini (2002)^5^. Following the specific determination of all collected specimens, they were incorporated into the Entomological Collection of the Oswaldo Cruz Institute, FIOCRUZ, titled ‘Atlantic Forest / RJ’.

This study employed various statistical analyses to comprehensively assess the mosquito community structure in each trap. The indices used included the Shannon diversity index (H), which measures species richness and evenness, with values starting at 0, where higher values represent higher diversity; equitability (J), which represents the evenness of species abundance and ranges from 0 to 1; species richness (S), which represents the total number of different species, the absolute abundance of individuals, reflecting the total number of mosquitoes; and the dominance index (D), which highlights patterns of dominance within a community and ranges from 0 to 1. The Morisita similarity index was used to quantify the degree of similarity between different mosquito breeding sites, as illustrated using a cladogram. This index helps to identify species distribution and composition patterns in different habitats. All statistical analyses were performed using the PAST software version 4.05, which provides a powerful platform for ecological data exploration.

The tire trap was the breeding site with the highest culicid density (n = 782). However, bamboo traps displayed the highest diversity index (H = 1.87), richness (S = 10), and equitability (J = 0.814). Sapucaia and tire traps showed similar diversity and richness indices (sapucaia traps: H = 1.17 and S = 6; tire traps: H = 1.16 and S = 7) (Table 1). The plastic container showed the lowest diversity index (H = 0.52) and the highest dominance (D = 0.741), with a predominance of *Aedes albopictus* Skuse, 1895 (85%). 

**TABLE 1:** Mosquito abundance and ecological indices per trap at the Fazenda dos Cordeiros, from March 2021 to October 2023.

Species	Tire-trap	Plastic-trap	Bamboo-trap	Sapucaia-trap
*Aedes (Protomacleaya) terrens*	147	4	30	32
*Aedes (Stegomyia) albopictus*	88	47	2	0
*Culex (Carrollia) iridescens*	60	4	33	14
*Culex (Microculex) pleuristriatus*	0	0	17	0
*Culex (Microculex) retrosus*	0	0	3	0
*Haemagogus leucocelaenus*	471	0	26	1
*Limatus pseudomethisticus*	5	0	0	0
*Toxorhynchites cf. grandiosus*	0	0	3	3
*Toxorhynchites cf. theobaldi*	0	0	0	2
*Toxorhynchites cf. pusillus*	2	0	0	3
*Trichoprosopon digitatum*	9	0	0	0
*Uranotaenia calosomata*	0	0	2	0
*Wyeomyia aporonoma*	0	0	2	0
*Wyeomyia (Phoniomyia) edwardsi*	0	0	26	0
**Total**	**782**	**55**	**144**	**55**
Species (S)	7	3	10	6
Dominance (D)	0.4168	0.7408	0.1765	0.4109
Shannon Diversity (H)	0.5968	0.4693	0.814	0.6553
Equitability (J)	0.5968	0.4693	0.814	0.6553

The plastic trap was the least frequent breeding site, hosting only three species: *Ae. terrens* Walker, 1856; *Ae. albopictus* Skuse, 1895, and *Culex iridescens* Lutz, 1905. The species found in all four analyzed breeding sites included *Ae. terrens* and *Cx.* (*Carrollia*) *iridescens*. Other species also had a high frequency and were recorded in three of the four analyzed breeding sites: *Ae. albopictus* and *Haemagogus leucocelaenus* Dyar and Shannon, 1924 (Table 1).

According to the Morisita similarity index, the most divergent breeding site was the plastic trap, with only three species recorded. The bamboo and sapucaia traps were the most similar breeding sites (Figure 2).

**FIGURE 2:**
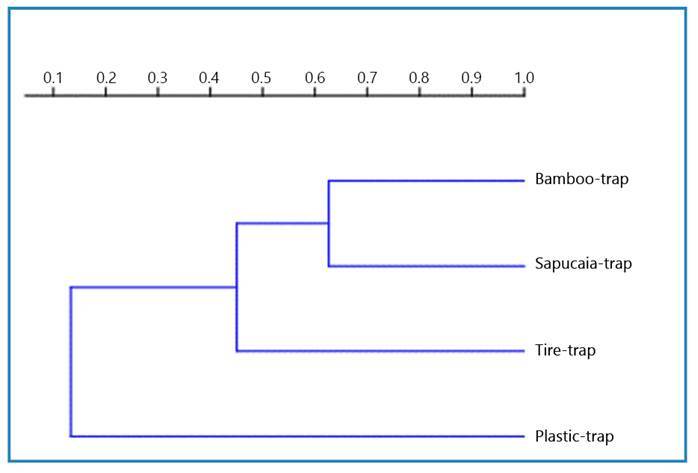
Morisita similarity index cladogram of mosquito breeding sites at Fazenda dos Cordeiros, municipality of Silva Jardim, Rio de Janeiro state, Brazil, from March 2021 to October 2023.

The broad range of breeding sites for culicid species is attributed to females’ choice of oviposition, which is a crucial component of their life cycle^4^. Different species exhibit distinct patterns of resource exploitation^3^. 

A study conducted in Curitiba, Paraná, Brazil reported that tire traps introduced into and adjacent to forest areas also collected immatures in greater quantity and frequency than other containers^8^. Tire traps are considered suitable breeding sites because of their concave shape, which facilitates water accumulation for longer periods, and their dark surface, which absorbs heat, creating a warmer and more attractive environment for mosquito larval proliferation. The species found exclusively in this trap was *Li. pseudomethisticus*. The presence of mosquito larvae in artificial containers highlights the ability of certain mosquito species to establish themselves in anthropogenically modified environments.

 Bamboo traps exhibited the highest diversity, richness, and equitability indices. Bamboo is a natural breeding site that is frequently used by culicids and other arthropods. This is primarily because of its culm structure, which facilitates water accumulation in its nodes and creates microhabitats with ideal conditions for the development of immature culicids^9^. The species found exclusively in this type of trap included *Uranotaenia calosomata*, *Wyeomyia aporonoma* and *Wy. (Phoniomyia) edwardsi*. A study conducted in fragments of the Atlantic Forest in Rio de Janeiro also reported the highest diversity index and elevated richness at this natural breeding site, with 15 culicid species documented^10^. In contrast, plastic traps were the least frequent breeding sites, with only *Ae. terrens*, *Ae. albopictus*, and *Cx. iridescens* found in this habitat. These genera and species exhibit high plasticity and adaptability to various habitats^11^ ^,^ ^12^. The plastic trap was the most divergent breeding site according to the Morisita similarity index. The most similar breeding sites were the bamboo and sapucaya traps; both bamboo and sapucaya are considered permanent or semi-permanent natural breeding sites. 

The species recorded at all the breeding sites were *Ae. terrens* and *Cx. iridescens*. The epidemiological importance of *Ae. terrens* can be attributable to elevated infection and dissemination rates this species has shown for Chikungunya virus (CHIKV) infection under experimental conditions^13^. Other species also exhibited a high frequency, being present in three of the four analyzed breeding sites: *Ae. albopictus* and *Hg. leucocelaenus*. Entomological investigations conducted during a yellow fever outbreak in southeastern Brazil from 2016 to 2019 identified *Hg. leucocelaenus* is the primary vector of Yellow Fever Virus (YFV), alongside *Hg. janthinomys* ^14^. *Aedes albopictus* is an effective vector of viruses that cause dengue and Zika, among other arboviruses^15^. 

The present results emphasize the importance of monitoring mosquito breeding sites in Atlantic Forest fragments, particularly those formed by bamboo internodes, given the diversity of recorded culicid species and the presence of those with epidemiological significance, such as *Hg. leucocelaenus*. It is crucial to highlight the role of bamboo in sustaining the large number of wild mosquito species in the biome. Breeding sites formed by bamboo are often overlooked in vector control strategies, posing potential risks to human populations residing in settlements near Atlantic Forest fragments. Given the crucial role of bamboo in sustaining mosquito populations, its inclusion is recommended in environmental management strategies aimed at reducing breeding sites for vector control in the Atlantic Forest. Such actions could involve the removal of bamboo from specific high-risk locations while still preserving the overall ecosystem balance.

## References

[1] WHO (2020). Vector-borne diseases.

[2] Alencar J, de Mello CF, Rodríguez-Planes L, dos Santos Silva J, Gil-Santana HR, Bastos AQ (2021). Ecosystem diversity of mosquitoes (Diptera: Culicidae) in a remnant of Atlantic Forest, Rio de Janeiro state, Brazil. Austral Entomol.

[3] Forattini OP (2002). Culicidologia Médica: Identificação, Biologia, Epidemiologia.

[4] Consoli R, Lourenço-de-Oliveira R (1994). Principais mosquitos de importancia sanitaria no Brasil.

[5] Chaves LSM, Rodrigues de Sá IL, Bergamaschi DP, Sallum MAM (2016). Kerteszia Theobald (Diptera: Culicidae) mosquitoes and bromeliads: A landscape ecology approach regarding two species in the Atlantic rainforest. Acta Trop.

[6] Bastos AQ, Leite PJ, de Mello CF, Maia DA, Machado SL, Gil-Santana HR (2021). Bionomy of Mosquitoes in Bamboo Internodes in an Atlantic Forest Remnant of the State of Rio De Janeiro, Brazil. J Am Mosq Control Assoc.

[7] Veloso H, Rangel-Filho A, Lima J (1991). Classificação da vegetação brasileira, adaptada a um sistema universal.

[8] Silva MAN da, Lozovei AL (1996). Criadouros de imaturos de mosquitos (Diptera, Culicidae) introduzidos em mata preservada na área urbana de Curitiba, Paraná, Brasil. Rev Bras Zool.

[9] Silva AM da, Nunes V, Lopes J (2004). Culicídeos associados a entrenós de bambu e bromélias, com ênfase em Aedes (Stegomyia) albopictus (Diptera, Culicidae) na Mata Atlântica, Paraná, Brasil. Iheringia Série Zool.

[10] Müller GA, de Mello CF, Bueno AS, de Alcantara Azevedo WT, Alencar J, Sharakhov IV (2022). Little noticed, but very important: The role of breeding sites formed by bamboos in maintaining the diversity of mosquitoes (Diptera: Culicidae) in the Atlantic Forest biome. PLoS One.

[11] Martins VEP, Alencar CHM de, Facó PEG, Dutra RF, Alves CR, Pontes RJS (2010). Distribuição espacial e características dos criadouros de Aedes albopictus e Aedes aegypti em Fortaleza, Estado do Ceará. Rev Soc Bras Med Trop.

[12] Lopes J (1997). Ecologia de mosquitos (Diptera: Culicidae) em criadouros naturais e artificiais de área rural do Norte do Estado do Paraná, Brasil. V. Coleta de larvas em recipientes artificiais instalados em mata ciliar. Rev Saude Publica.

[13] Lourenço-de-Oliveira R, Failloux AB (2017). High risk for chikungunya virus to initiate an enzootic sylvatic cycle in the tropical Americas. PLoS Negl Trop Dis.

[14] de Abreu FVS, Ribeiro IP, Ferreira-de-Brito A, dos Santos AAC, Miranda RM, Bonelly IS, Neves MSAS, Bersot MI, Santos TP, Gomes MQ (2019). Haemagogus leucocelaenus and Haemagogus janthinomys are the primary vectors in the major yellow fever outbreak in Brazil, 2016-2018. Emerg Microbes Infect.

[15] Gloria-Soria A, Payne AF, Bialosuknia SM, Stout J, Mathias N, Eastwood G (2020). Vector Competence of Aedes albopictus Populations from the Northeastern United States for Chikungunya, Dengue, and Zika Viruses. Am J Trop Med Hyg.

